# Editorial: Aortic diseases involving visceral artery: clinical therapeutic management and related basic research

**DOI:** 10.3389/fcvm.2024.1449444

**Published:** 2024-07-03

**Authors:** Xiangjiu Ding, Mian Wang

**Affiliations:** ^1^ Department of Vascular Surgery, General Surgery, Qilu Hospital of Shandong University, Jinan, China; ^2^ Department of Vascular Surgery, General Surgery, Qilu Hospital of Shandong University, Qingdao, China; ^3^ Division of Vascular Surgery, The First Affiliated Hospital of Sun Yat-sen University, Guangzhou, China

**Keywords:** aortic diseases, visceral artery, treatment, endovascular, surgery

**Editorial on the Research Topic** Aortic diseases involving visceral artery: clinical therapeutic management and related basic research

## Introduction

Aortic diseases involving visceral arteries mainly include true and post-dissection thoracoabdominal or abdominal aortic aneurysm (TAAA or AAA). Post-dissection aortic aneurysm is common in both type A and type B aortic dissection, even after successful coverage of the primary entry tears. False aortic aneurysm involving visceral arteries is relatively uncommon. The management of these complex aortic diseases is usually challenging due to involvement of visceral arteries. Several surgical treatment methods are used for these diseases, mainly including open surgical repair, hybrid surgery, and endovascular treatment.

## True TAAA or AAA

Standard open repair for TAAA or AAA involving visceral arteries was usually associated with definite effect, large trauma, and high mortality and morbidity. With the development of surgical techniques and perioperative adjunctives, many studies suggest clinical results have been significantly improved in high-volume hospitals (1, 2). The in-hospital mortality was significantly decreased in the past three decades, as well as the rate of permanent spinal cord ischemia (2). Open surgical repair remains a valid treatment option for patients with long life expectancy.

In 1999, Quinones-Baldrich et al. first reported the hybrid surgery for a type IV TAAA (3). It involves sequentially bypassing the visceral arteries and using an uninvolved vessel for inflow to disconnect them from the aneurysmal aorta. Mortality after hybrid surgery is highly variable by center, but strongly affected by preoperative comorbidities and the centers’ experience with the technique (4).

Ma et al. compared clinical outcomes after hybrid surgery with conventional open surgery. Although the age of the hybrid surgery group was significantly higher than that of the other group, perioperative mortality was low in the hybrid surgery group compared with conventional surgery group. Furthermore, the rates of postoperative complications such as renal failure, respiratory failure, and deep venous thrombosis following hybrid surgery were significantly lower than conventional open surgery. Hybrid surgery is technically feasible and associated with definite efficacy in selected cases. It simplifies the operation procedure and reduces the risks of mortality and morbidity in high-risk or high-age patients. Hybrid surgery may be a promising alternative to conventional open surgery in selected patients.

Endovascular aortic repair (EVAR) is associated with small trauma and rapid recovery. It mainly includes parallel stenting technique and fenestrated/branched endografts to preserve the visceral arteries. The use of parallel stenting is limited due to high risk of type IA endoleak, and the risk of stent collapse. The joint use covered stent graft and overlapped bare stents in the visceral artery segment demonstrated favorable mid-term clinical outcomes in type V TAAAs (5). Using of bifurcated abdominal aortic stent graft main body and docking two or three parallel covered stents in the short limb for the reconstruction of visceral arteries, known as “Octopus” technique, demonstrated promising mid-term outcomes for treating ruptured and symptomatic TAAAs (6).

Branched or fenestrated endograft can be custom-made, off-the-shelf, or physician-modified. For custom-made endografts, the manufacturing time limits their use. The shortcomings of physician-modified endografts include technical demanding, potential contamination risk, and damage of device integrity, as well as legal issues. A recent meta-analysis suggested the use of the off-the-shelf t-Branch multibranched endograft for endovascular TAAA repair was associated with high technical success rates and demonstrated to be safe and effective at early and mid-term follow-up (7). The primary use of a novel G-Branch multibranched (two inner branches and two outer branches) endograft also yielded good early and midterm outcomes (8). Treatment modality of TAAA or AAA involving visceral arteries is gradually shifted from conventional open surgery to endovascular repair in many countries (9).

## PD-TAAA or PD-AAA

Studies with regard to open surgical repair for PD-TAAA or PD-AAA involving visceral arteries were relatively few in literature. Limited data showed conventional open surgery was more invasive than endovascular treatment, while it was also associated with acceptable rates of morbidity and mortality when it was performed in a specialized hospital. Long-term results were excellent and it should also be considered when evaluating less invasive alternatives (10). More studies are required to verify its safety and efficacy.

Post-dissection aortic aneurysms are increasingly being treated by endovascular repair. A large number of endovascular methods have been reported. Fenestrated/branched-EVAR (F/B-EVAR) is the most commonly used method (11). Other methods are usually associated with a small number of cases. F/B-EVAR can cover multiple tears regardless of sizes and preserve branch organ perfusion. However, the endografts usually need to be modified by physicians. It is technically demanding, which limits its widespread use. Spinal cord ischemia, endoleak, and stent or stent-graft occlusion of branch arteries are common complications. Other substitution methods are currently explored.

F/B-EVAR mainly focuses on the management of the true lumen, while management of false lumen and aortic tears has been attempted (12). Isolated management of the false lumen or tears seems to make it difficult to achieve satisfactory outcomes for PD-TAAA or PD-AAA (13). Several combined methods have been reported. They usually combine the management of two or three objectives (true lumen, false lumen, and aortic tears), such as “road block” strategy, “double splints” technique, and spot stenting combined with false lumen endovascular occlusive repair (14–16). The preliminary results of these techniques were acceptable, despite of the requirement for higher-grade evidence. One of the most important problems is how to occlude the false lumen and the tears safely and effectively. There have been no available specialized devices to date. New novel devices are anticipated to help resolve this issue in future, such as EndoSeal and Endopatch system (17, 18). Visceral arteries arising from the false lumen can be reconstructed with direct stent-graft placement via adjacent tears, reverse branch technique, iliac branched device, or *in situ* fenestration.

In conclusion, endovascular treatment is gradually become the mainstream treatment for true or post-dissection TAAA or AAA involving visceral arteries. Open surgery and hybrid surgery still are valid treatment options for selected patients, such as low-risk patients with long-life expectancy and those unfit for endovascular repair.
